# Nurses' Knowledge and Attitude towards Postoperative Pain Management in Ghana

**DOI:** 10.1155/2020/4893707

**Published:** 2020-08-07

**Authors:** Shamsu-Deen Mahama Adams, Shokoh Varaei, Fatemeh Jalalinia

**Affiliations:** School of Nursing and Midwifery, Tehran University of Medical Sciences, International Campus, Tehran, Iran

## Abstract

**Background:**

Pain management is a very important aspect of nursing care among postoperative patients. Deficit in the knowledge and bad attitude towards pain management among nurses remain a problem in Ghana. In order to manage pain better in the surgical wards, nurses should be well equipped with knowledge of pain assessment and management.

**Purpose:**

The purpose of the study was to determine nurse's knowledge and attitude towards pain management among postoperative patients in surgical units in Ghana. *Methodology*. This study used the quantitative study approach with a descriptive cross-sectional study design. A sample of 211 nurses was recruited using the convenience sampling method. Data were collected using a questionnaire regarding postoperative pain management. Descriptive statistics, Pearson's correlation coefficient, and the chi-squared test were used to analyze the data using SPSS version 16.0.

**Results:**

The mean age of the nurses was 29.77, with the youngest nurse being 23 years and oldest being 39 years. Majority (72.5%) of nurses had moderate knowledge, and 89.6% of the nurses had negative attitude towards pain management. There was no significant relationship between nurse's knowledge and years of experience as a nurse (r = −0.03, *p*=0.64), as well as no significant relationship between knowledge and number of years working in the surgical ward (r = 0.06, *p*=0.36). Also, there was no significant relationship between nurses' knowledge and nurses' attitude (r = 0.06, *p*=0.36). *Conclusion and recommendation*. The level of knowledge and attitude towards postoperative management were generally inadequate among nurses. Therefore, there is the need to implement in-service training on pain management for nurses working in the surgical units frequently.

## 1. Introduction

Pain is an unpleasant emotional or sensory experience associated with actual or potential damage to the tissues [1]. The most important factor in determining appropriate and effective pain management in the concept is that, pain is a completely unique and subjective human perception and experience [2]. Postsurgical/postoperative pain has been described as a complex response to tissue trauma during surgery that stimulates an aversion of the central nervous system. It is experienced immediately after surgery when the anesthesia effect is worn off [3]. Pain is one of the most common reasons why people seek healthcare and be admitted into hospitals, and it accounts for two-thirds of visits to the emergency units in the United States [4].

A study in India by Sommer et al. reported a postoperative experience of moderate to severe pain by 41% among respondents of 1,490 postoperative patients [5].

In another study, Zaslansky et al. surveyed about 6,000 adult patients in orthopedic and general surgery in Europe and Israel. The results revealed that 70% and 48% of the postoperative patients reported moderate and severe postoperative pain, respectively [6].

Postoperative pain is described as a common clinical condition that when poorly controlled can result in a number of significant negative consequences to the patient. Pain is among the most fearsome symptoms many people fear, and if untreated, it can have many negative consequences. [7]. Despite significant advances in technology and medications, unrelieved postoperative pain continues to be problematic for surgical patients as statistics indicate that about 43 million patients in the United States experience acute postoperative pain, with pain intensities of moderate to severe reported by 80% of these patients. Additionally, about 50% of postoperative patients report unrelieved pain [8].

Unrelieved pain causes discomfort that deprives individuals sleep, causes depression, increases anxiety, leads to morbidity, and eventually death [9].

According to Ayla Yava et al., in a study conducted in Turkey, patients still experience unnecessary pain in many hospitals, especially postoperatively despite the growing awareness on pain management in many health institutions. The nurse plays an important role in the effective management of postoperative pain because it is the responsibility of the nurse to assess pain and provide a timely intervention [10]. Many disciplines are involved in pain management; however, nurses play a pivotal role in the assessment, relief, and evaluation of pain. Pain experience is a universal one, subjected to individual interpretation. It manifests in several ways, which are very difficult to accurately assess [11].

The assessment and management of acute postoperative pain is important in the care of postoperative patients. Nurses are always exposed to patients experiencing some degree of pain and should be knowledgeable about how to care for those patients. Management of postoperative pain reliefs suffering and leads to earlier mobilization, shortened hospital stay, reduced hospital costs, and increased patient satisfaction [12]. Research findings suggested that nurses underestimates patient's pain, do not believe that patients have pain, and do not administer the prescribed dosage of analgesics for the fear of addiction. Nurses who care for postoperative patients must recognize the need for adequate pain management and needs expertise in pain management and the use of other techniques in pain management such as patient-controlled analgesia [13]. Eventhough the problem has been researched in many hospitals around the world, no known research has been carried out in this current location of the country.

Effective postoperative pain management is one of the most important aspects of patient management after surgery. Nurses have the responsibility to effectively manage pain; as a result, there is the need for them to have requisite knowledge and skills about pain management [14].

When pain is not effectively managed, it brings about physical and emotional stress responses, which inhibit healing, increase the risk for other complications, and increase the length of stay in the critical care. Pain is one of the most common problems faced by nurses when dealing with patients in the surgical units [15].

## 2. Nurses' Knowledge and Attitude regarding Postoperative Pain Management

Based on the up-to-date knowledge, a positive attitude and a good practice of pain management by the nurse will minimize the consequence and complications of pain; as a result, the nurse is obliged to possess an updated knowledge and understanding of pain.

Studies have shown that nurses lack adequate knowledge and attitude towards effective postoperative pain management and also their inability to effectively assess and manage pain [16]. A study conducted in Zimbabwe shows that nurses had inadequate knowledge with a mean knowledge score of 64.5%, and attitude regarding pain management of adult medical patients was average with a total mean attitude score of 56% [17]. Also findings from a study conducted in Cairo to determine critical care nurses' knowledge and practices regarding pain assessment and management at Cairo University' Hospitals show that majority of the nurses (93.3% and 95%) had an unsatisfactory knowledge and practices level, respectively [18].

Results of a study conducted in Uganda determine nurses' knowledge and practices related to pain assessment in critically ill patients reveal that majority of the nurses (91.2%) had adequate knowledge. Also, about half lacked knowledge on key pain assessment principles, 43.5% did not state patient as the most accurate in rating the pain intensity, and 44% of nurses seldom agree with what patients' say about pain [19].

The study of Aziato (2013) in Ghana reveals that Ghanaian nurses' inadequate pain management knowledge might have resulted from curriculum gaps during training; inadequate clinical supervision, study days, and workshops for practicing nurses; lack of funding for organizing regular workshops; and negative attitude of nurses whereby new information learned at workshops was not readily applied in clinical practice [20].

### 2.1. Purpose

The purpose of the study was to determine nurse's knowledge and attitude towards effective postoperative pain management in Ghana.

## 3. Methods

### 3.1. Study Design

A descriptive cross-sectional study was used in this study. This was to enable the researcher to systematically determine and report the level of knowledge and attitude of nurses towards effective postoperative pain management.

### 3.2. Study Setting

The study was conducted at the surgical units of the Tamale Teaching Hospital in the Northern regional capital of Ghana. It serves as the referral center for all the district hospitals and health centers throughout the three northern regions (Northern, Upper East, and Upper West) and parts of the Brong Ahafo region. The medical staff comprises 76 doctors and 70 house officers and 890 nurses of all categories. It has an in-patient bed capacity of 483 beds.

### 3.3. Sample and Sampling Method

250 registered nurses were recruited from various surgical units of the hospital. A total of 211 respondents completed their questionnaire out of the eligible 250 (84.4% response rate).

The study utilized the convenience sampling method in recruiting the nurses into the study. The nurses were approached at the time of the study at the selected units, explained the purpose of the study, and requested for their participation.

### 3.4. Instrument and Procedure

A modified version of the knowledge and attitude survey regarding pain was used to collect data to suit the current study by Farrel and McCaffery [20]. The questionnaire was developed specifically to suit the study after permission was granted by the developers. It consists of 28 true/false and multiple choice knowledge questions and 9 true/false and multiple choice attitude questions. A knowledge score of 70% and above was regarded as adequate knowledge and a desirable score for this study, and an attitude score of 70% and above was regarded as positive attitude and a desirable score for this study. After an informed consent was obtained from nurses and based on inclusion criteria, the questionnaires about knowledge and attitude towards pain management were distributed among the nurses. After completion, the questionnaires were then picked up from various surgical units. The questionnaire was given as and when a respondent was available. It took two months for collection of data (July and August).

### 3.5. Ethical Consideration

Permission to carry out the study was sought from Research ethical committee of Tehran University of Medical Science (TUMS) international campus (IR.TUMS.FNM.REC.1396.2695) and the Research and Ethics committee of Tamale Teaching Hospital (TTH) (TTH/R&D/17/85), as well as the Nursing Administration of the hospital. Informed consent was also signed by participants.

### 3.6. Data Analysis

The data analysis was performed using the Statistical Package for Social Sciences (SPSS) version 16.

The data analysis included descriptive statistics, using frequencies, mean, and standard deviation. Data were further examined using correlational statistics to explore the relationship between variables.

## 4. Results

### 4.1. Demographic Data

The respondents were in an age category of 23–39 years. The mean age was 29.7 years with a standard deviation of 3.9 years. The respondents had 1–16 years of experience as a nurse and 1–10 years as a nurse in surgical ward (Table 1). Majority of the respondents (122 (57.8%)) were females. There were 131 (62.1%) diploma holders and 69 (32.7%) staff nurses. The majority of the respondents (130 (61.6%)) have never received training on pain management (Table 2).

The correctly answered knowledge-related questions by participants ranged from 19.9% to 92.9%. Among all questions, question 22 received the highest correct responses (92.9 %), and question 12 received the lowest correct responses (19.9%) (Table 2).

The total mean knowledge score was 59% with a range of 25–82%. The majority of the respondents (167 (79.1%)) had inadequate knowledge, while 44 (20.9%) had adequate knowledge towards postoperative pain management..

### 4.2. Attitude towards Pain

The correctly answered attitude-related questions by participants ranged from 12.8% to 83%. Among all questions, question 6 received the highest positive response (83.4%), and question 7 received the lowest positive responses (12.8%). 162 (76.8%) were not able to identify that patient may sleep in spite of pain. Also, 124 (58.8%) fail to identify that patients who can be distracted from pain usually do not have severe pain. 184 (87.2%) were also not able to identify that giving patients sterile water by injection (placebo) was not a useful test to determine if the pain was real. In all, question 6 had the correct rate greater than 70%, and 8 questions had correct rates below 70 % (Table 3).

The total mean attitude score was 52% with a range of 22–89%. The majority of the respondents (189 (89.6%)) had negative attitude, while 22 (10.4%) had positive attitude towards postoperative pain management.

## 5. Discussions

The participants had a mean age of 29.77 ± 3.96; the youngest participant was 23 years, and the oldest was 39 years. This may be as a result of older nurses taking up management positions making it difficult for them to be involved in pain management. This finding was supported by the findings of D'emeh et.al in a study to determine pain-related knowledge and barriers among Jordanian nurses [21]. They found out that majority of the nurses who responded aged between 18 and 25 years (33.7%) and between 26 and 35 years (27.6%). Majority of the participants were females and also most were married. This finding might be as a result of a perception in Ghana that the nursing profession was meant for females because its origin dates back to Florence Nightingale. This was consistent with a study finding by Kizza et al. . They found out that majority of the nurses were females [22].

Regarding the level of education, majority of the nurses had only diploma (62.1%) with the rest attaining bachelor's degrees and master's degrees in nursing. The reason might be because diploma is the minimum degree needed to be able to practice as a registered general nurse in Ghana. This was consistent with the findings of Hossain who reported in his study that majority of the nurses had only Diploma (80.6%) [23].

Most of the nurses were staff nurses with some being senior staff nurses, nursing officers, senior nursing officers, and principal nursing officers. The findings of this research in this regard may be as a result of nurses serving for longer years before they are promoted. In Ghana, it takes 2 and 4 years for a staff nurse to be promoted to senior staff nurse and from senior staff nurse to nursing officer respectfully. The findings were, however, inconsistent with the findings of Hossain, which show that majority of the nurses are senior staff nurses. With regards to receiving training on effective pain management, majority of the nurses have never received training on effective pain management [23]. This might be because the management of the hospital rarely organized workshops for nurses as they depend on donor partners and nongovernmental organizations for workshops, which occur once in a blue moon. This finding was consistent with the findings of Basak . He found out that all nurses who participated in the study have never received training on effective pain management [24].

Regarding the level of nurses' knowledge towards postoperative pain management, the average nurses' knowledge was inadequate.

Findings from the study revealed that majority of the nurses (79.1%) had inadequate knowledge. Participants had only 20.9% adequate knowledge. The inadequate knowledge in this current study might be because majority of the participants were diploma nurses; as a result, pain management is not included their curriculum. This group of nurses gains knowledge on pain management from activities of their senior colleagues and doctors. The findings were supported by Kaur in a study to assess the knowledge and attitude of staff nurses regarding pain management at Kular Hospital, Bija (Punjab). His findings revealed that majority of the respondents (66%) had moderate knowledge [25].

However, the findings of this study were inconsistent with the findings of Matthews and Malcolm in their study of nurses' knowledge and attitude regarding pain management in Northern Ireland. They found a score of 73.8%, which was more favorable than this present study [26].

Similarly, the findings of this study contradicted with the findings of Ali et al. who found out in their study that nurses have satisfactory knowledge [27].

The study also found out that nurses demonstrated a poor level of knowledge in important areas of pain management such as pharmacology. This may be because pharmacology is inadequately covered at the diploma level, and since majority of the nurses were diploma holders and never attended any pain management training, they had poor pharmacological knowledge. This finding was supported by the findings of Lewthwaite et al. in a descriptive study in an urban tertiary care hospital to explore the knowledge and attitude of registered nurses regarding pain management [28]. They found out that there was poor pharmacology knowledge among nurses regarding postoperative pain management. Some of the incorrectly answered pharmacology questions were as follows: aspirin and other nonsteroidal anti-inflammatory agents are not effective analgesics for acute postoperative pain (68.7%), and the usual duration of analgesia of 1-2 mg morphine IV is 4-5 hours (66.4%). Opioid should not be used in patients with a history of substance abuse (78.2%). Anticonvulsant drugs such as gabapentin (Neurontin) produce optimal pain relief after a single dose (68.7%).

The current findings of this study also reveal that majority (89.6%) of the nurses had negative attitude towards postoperative pain management. Nurses had only 10.4% adequate attitude. This finding might be as a result of the belief in Ghana that pain is necessary in order to be healed. Most nurses might be harboring this belief since majority of the nurses in this study have never received any training on pain management, which would have helped to know that effective pain management is crucial to complete recovery of a postoperative patient.

The study findings were consistent with the findings of Basak who found out in his study that most nurses had negative attitude towards postoperative pain management [24]. The finding was also supported by findings of Issa et al. in their study [29]. They found out that ICU nurses had negative attitude towards pain management. Eventhough nurses answered most of the attitude questions correctly, it was not up to the cut point of 70% for a positive attitude. Some of the answered attitude questions are as follows: patients who can be distracted from pain usually do not have severe pain (41.2%), patient may sleep in spite of pain (23.2%), elderly patients cannot tolerate opioid for pain relief (61.1%), patients should be encouraged to endure as much pain as possible before using an opioid (57.3%), and children less than 11 years old cannot reliably report pain, so clinicians should rely solely on the parents of the child for pain intensity (69.7%) etc.

There was no significant relationship between nurses' years of experience as a nurse and knowledge (*r* = −0.03, *p*=0.64), as well as no significant relationship between nurses' years of experience as a nurse in surgical ward and knowledge (*r* = 0.06, *p*=0.36). These findings were consistent with the findings of Basak, in a study, to determine knowledge and attitude among nurses and their practices regarding postoperative pain management in Bangladesh [24].

However, the findings were inconsistent with the findings of Lui et al. They found out that nurses' knowledge scores increased markedly with increased work experience in a study to determine knowledge and attitude regarding pain management among nurses in Hong Kong [30].

The findings also show no significant relationship between knowledge and attitude (*r* = 0.06, *p*=0.36).This was consistent with the study of Basak who also found no statistical significant relationship between knowledge and attitude (*r* = 0.16, *p* > 0.05) [24].

## 6. Conclusion

The purpose of the study was to determine nurse's knowledge and attitude towards effective postoperative pain management in Ghana. The results of this study support the universal concern of inadequate knowledge and negative attitude among nurses in Ghana.

The findings of the study revealed a moderate level of nurses' knowledge and negative attitude of nurses towards postoperative pain.

## 7. Strengths and Limitations of the Study

This study offers evidence to improve upon postoperative pain management in Ghana, even though it was conducted in one educational hospital. The researcher was able to recruit nurses from different kinds of ranks/positions from the hospital to give a true representation of the study population.

Apart from the strengths of the study, there were limitations. First, this study was conducted in one educational hospital in Tamale, a city in Ghana. The educational hospital in Tamale may not be comparable to the other two educational hospitals in Ghana; therefore, this might limit the generalization of the findings, especially due to the small sample size used in the study. Second, the study used a simple method to collect data, a self-report. This method has a limitation in itself, especially when it is used to rate human actions.

## 8. Recommendation

It is evident from the results of this study that surgical nurses at the Tamale Teaching Hospital are not up-to-date with the current literature on postoperative pain management as it has manifested in their practice.

Knowledge about pain, pain assessment, and pharmacological and nonpharmacological pain management should be added as content to the curriculums for diploma and bachelor nursing students.

An in-service education and training in postoperative pain management should be carried out on regular basis to update nurses on the current trends in postoperative pain management.

Furthermore, qualitative and experimental research in specific areas of postoperative pain management is also recommended.

## Figures and Tables

**Table 1:** Demographic data (*n* = 211). Knowledge of pain management.

Variables	Minimum	Maximum	Mean ± SD

Age (Years)	23	39	29.77 ± 3.952

Experience as a nurse (years)	1	16	4.33 ± 2.540

Experience as a nurse in surgical	1	10	2.67 ± 1.682

Ward (years)			
	Frequency	Percent	

Gender			
Male	89	42.2	
Female	122	57.8	

Marital status			
Married	116	55.0	
Single	95	45.0	

Level of education			
Diploma	131	62.1	
Undergraduate	77	36.5	
Masters	3	1.4	

Rank (position)			
Staff nurse	69	32.7	
Senior staff nurse	57	27.0	
Nursing officer	56	26.5	
Senior nursing officer	28	13.3	
Principal nursing officer	1	0.5	

Training on pain			
Yes	81	38.4	
No	130	61.6	

Total	211	100.0	

**Table 2:** Knowledge of pain management (211).

Variable	Correct	Incorrect
(1) Vital signs are always reliable indicators of the intensity of a patient's pain	77 (36.5%)	134 (63.5%)
(2) Because their nervous system is underdeveloped, children under two years of age have decrease pain sensitivity and limited memory of painful experiences	69 (32.7%)	142 (67.3%)
(3) Aspirin and other nonsteroidal anti-inflammatory agents are not effective analgesics for acute postoperative pain	66 (31.3%)	145 (68.7%)
(4) Respiratory depression rarely occurs in patients who have been receiving stable doses of opioid over a period of months	118 (55.9%)	93 (44.1%)
(5) Combining analgesics that work by different mechanisms may result in better pain control with fewer side effects than using a single analgesic agent	163 (77.3%)	48 (22.7%)
(6) The usual duration of analgesia of 1-2 mg morphine IV is 4-5 hours	71 (33.6%)	140 (66.4%)
(7) Pethidine 75 mg IM is approximately equal to morphine 10 mg IM	121 (57.3%)	90 (42.7%)
(8) Opioid should not be used in patients with a history of substance abuse	46 (21.8%)	165 (78.2%)
(9) The term “equianalgesia” means approximately equal analgesia	187 (88.6%)	24 (11.4%)
(10) After an initial dose of opioid analgesic is given, subsequent doses should be adjusted	189 (89.6%)	22 (10.4%)
(11) Anticonvulsant drugs such as gabapentin (Neurontin) produce optimal pain relief after a single dose	66 (31.3%)	145 (68.7%)
(12) If the source of the patient's pain is unknown, opioid should not be used during the pain evaluation period	42 (19.9)	169 (80.1%)
(13) Sedation assessment is recommended during opioid pain management	176 (83.4%)	35 (16.6%)
(14) Benzodiazepines are not effective pain relievers and are rarely recommended	158 (74.9%)	53 (25.1%)
(15) Narcotic/Opioid addiction is defined as a chronic neurobiological disease, characterized by behaviors that include one or more of the following: impaired control over drug use, compulsive use, continued use despite harm, and craving	191 (90.5%)	20 (9.5%)
(16) The recommended route of administration of opioid analgesics for patients with persistent postoperative pain is	84 (39.8%)	127 (60.2%)
(17) The recommended route of administration of opioid analgesics for patients with brief, severe pain of sudden onset is	145 (68.7%)	66 (31.3%)
(18) Which of the following analgesic medications must be used with caution due to its metabolite that can precipitate seizures?	105 (49.8%)	106 (50.2%)
(19) A 30 mg dose of oral morphine is approximately equivalent to	132 (62.6%)	79 (37.4%)
(20) Analgesics for postoperative pain should initially be given	185 (88.2%)	25 (11.8%)
(21) A patient with severe postoperative pain has been receiving daily morphine injections for 3 consecutive days. The likely hood of the patient developing clinical psychological addiction is	104 (49.3%)	107 (50.7%)
(22) Which of the following nondrug methods are useful for combining with treatment of postoperative pain?	196 (92.9%)	15 (7.1%)
(23) The most accurate judge of the intensity of the patient's pain is	180 (85.3%)	31 (14.7%)
(24) How likely is it that those patients who develop pain already have alcohol and/or drug abuse problem?	118 (55.9%)	93 (44.1%)
(25) The time to the peak effect for morphine given IV is	145 (68.7%)	66 (31.3%)
(26) The time to the peak effect for morphine given orally is	138 (65.4%)	73 (34.6%)
(27) Following abrupt discontinuation of an opioid, physical dependence is manifested by the following:	91 (43.1%)	120 (56.9%)
(28) Which statement is true regarding opioid-induced respiratory depression	105 (49.8%)	106 (50.2%)

**Table 3:** Attitude towards pain (211).

Variable	Correct	Incorrect
(1) Patients who can be distracted from pain usually do not have severe pain	87 (41.2%)	124 (58.8%)
(2) Patient may sleep in spite of pain	49 (23.2%)	162 (76.8%)
(3) Elderly patients cannot tolerate opioid for pain relief.	129 (61.1%)	82 (38.9%)
(4) Patients should be encouraged to endure as much pain as possible before using an opioid	121 (57.3%)	90 (42.7%)
(5) Children less than 11 years old cannot reliably report pain so clinicians should rely solely on parents for the child's pain intensity	147 (69.7%)	64 (30.3%)
(6) Patients' spiritual beliefs may lead them to think pain and suffering are necessary	176 (83.4%)	35 (16.6%)
(7) Giving patients sterile water by injection (placebo) is a useful test to determine if the pain is real	27 (12.8%)	184 (87.2%)
(8) The most likely reason a patient with pain would request increased doses of pain medication is	129 (61.1%)	82 (38.9%)
(9) Which of the following describes the best approach for cultural considerations in caring for patients in pain?	128 (60.7%)	83 (39.3%)

## Data Availability

Data were collected from nurses at the Tamale teaching hospital after permission was granted.
